# Usefulness of health registries when estimating vaccine effectiveness during the influenza A(H1N1)pdm09 pandemic in Norway

**DOI:** 10.1186/1471-2334-12-63

**Published:** 2012-03-20

**Authors:** Bernardo Rafael Guzmán Herrador, Preben Aavitsland, Berit Feiring, Marianne A Riise Bergsaker, Katrine Borgen

**Affiliations:** 1 Division of Infectious Disease Control, Norwegian Institute of Public Health, Oslo, Norway; 2 European Programme for Intervention Epidemiology Training (EPIET), European Centre for Disease Prevention and Control (ECDC), Stockholm, Sweden

## Abstract

**Background:**

During the 2009-2010 pandemic in Norway, 12 513 laboratory-confirmed cases of pandemic influenza A(H1N1)pdm09, were reported to the Norwegian Surveillance System for Communicable Diseases (MSIS). 2.2 million persons (45% of the population) were vaccinated with an AS03-adjuvanted monovalent vaccine during the pandemic. Most of them were registered in the Norwegian Immunisation Registry (SYSVAK). Based on these registries, we aimed at estimating the vaccine effectiveness (VE) and describing vaccine failures during the pandemic in Norway, in order to evaluate the role of the vaccine as a preventive measure during the pandemic.

**Methods:**

We conducted a population-based retrospective cohort study, linking MSIS and SYSVAK with pandemic influenza vaccination as exposure and laboratory-confirmed pandemic influenza as outcome. We measured VE by week and defined two thresholds for immunity; eight and 15 days after vaccination.

**Results:**

The weekly VE ranged from 77% to 96% when considering 15 days or more after vaccination as the threshold of immunity and from 73% to 94% when considering eight days or more. Overall, 157 individuals contracted pandemic influenza eight or more days after vaccination (8.4/100,000 vaccinated), of these 58 had onset 15 days or more after vaccination (3.0/100,000 vaccinated). Most of the vaccine failures occurred during the first weeks of the vaccination campaign. More than 30% of the vaccine failures were found in people below 10 years of age.

**Conclusions:**

Having available health registries with data regarding cases of specific disease and vaccination makes it feasible to estimate VE in a simple and rapid way. VE was high regardless the immunity threshold chosen. We encourage public health authorities in other countries to set up such registries. It is also important to consider including information on underlying diseases in registries already existing, in order to make it feasible to conduct more complete VE estimations.

## Background

During the 2009-2010 influenza pandemic, surveillance for influenza in Norway was enhanced: laboratory-confirmed infection with the new A(H1N1)pdm09 virus was defined as a mandatory notifiable disease on a case based level. During the pandemic, doctors from all health care settings were encouraged to swab patients with suspected influenza and submit the samples to their nearest clinical microbiology laboratory. Each laboratory-confirmed case had to be notified to the Norwegian Institute of Public Health (NIPH), where it was registered in the Norwegian Surveillance System for Communicable Diseases (MSIS) with the unique personal identification number (PIN). Criteria for sampling suspected cases changed during the pandemic. During the first months of the pandemic (from May until October 2009) all suspected cases were sampled. However, as the pandemic developed and number of cases increased, only those that belonged to risk groups or with severe influenza like illness (ILI) symptoms were sampled since it was no longer feasible to swab all suspected cases. In total, 12,513 influenza A(H1N1)pdm09 laboratory-confirmed cases were registered in MSIS during the pandemic (May 2009-April 2010). The first scattered cases were recorded in early May 2009 among travellers returning from countries where the virus was already circulating. In July and August (weeks 26-35) a first wave occurred. The second and main wave during October and November (weeks 43-49) reached a higher peak. From December the number of cases decreased until April 2010, when the last cases were reported [1].

On 19 October 2009, just before the main wave of the pandemic, the vaccination campaign against influenza A(H1N1)pdm09 started in Norway with vaccine available from GlaxoSmithKline Biologicals S.A. (GSK) through an advance purchase agreement. Some days before, on 25 September 2009, the European Medicines Agency (EMA) had recommended marketing authorisation of GSK's AS03-adjuvanted monovalent vaccine containing 3.75 micrograms of hemagglutinin antigen per dose [2]. The vaccine contained the influenza strain A/California/7/2009 (H1N1) [3]. At the beginning, two doses were recommended by EMA based on data on the mock up vaccine, containing an A(H5N1) virus strain [4]. However, clinical trials containing A(H1N1)pdm09 indicated that one dose would provide sufficient immunogenicity in most formulations [2] and the recommendation was therefore changed at the end of October 2009. At the beginning of the vaccination campaign in Norway only health care workers, pregnant women and children and adults with certain underlying diseases were recommended to be vaccinated. The recommendation was expanded to the whole population over six months of age on 23 October 2009 [5,6].

The Norwegian Immunisation Registry (SYSVAK), where all the vaccinations given in the childhood immunisation programme in Norway have routinely been registered since 1995, was adapted for registration of vaccination against A(H1N1)pdm09 during the pandemic. A new Internet based registration module with automatic linkage to the national population registry containing the PIN of all individuals in Norway was created. The Ministry of Health and Care Services decided in October 2009, just before the start of the vaccination campaign, that there should be mandatory notification to SYSVAK of all vaccinations against pandemic influenza. An estimate by the Norwegian health authorities indicated that 2.2 million people (45% of the total population) were vaccinated during the campaign [7]. Most of them, 1,963,895 persons, were notified to SYSVAK.

We aimed to estimate the vaccine effectiveness (VE) of the AS03-adjuvanted monovalent vaccine (from now called "pandemic influenza vaccine") against laboratory-confirmed influenza A(H1N1)pdm09 and describe the individuals who had laboratory-confirmed influenza in spite of vaccination (vaccine failures) during the 2009-2010 influenza pandemic in Norway.

## Methods

We conducted a population-based retrospective cohort study during the main wave of the pandemic, from October 2009 until December 2009.

Laboratory-confirmed cases of A(H1N1)pdm09 influenza registered in MSIS were linked to their vaccination status in SYSVAK by the unique PIN. Both registries were created for surveillance purposes and are managed by the Division of Infectious Disease Control at the NIPH. Ethical approval was not required as updating of MSIS with the patients' vaccination histories from SYSVAK is part of the quality assurance of MSIS.

For analysis the following variables of interest were extracted from MSIS: sex, county of residence, year of birth, date of onset of influenza and date of laboratory testing. For those cases in which the real date of onset of symptoms was unknown, a proxy date of onset was estimated by subtracting two days (average of days between date of onset and date of laboratory testing in those cases in which both real dates were known) from the date of laboratory testing. The date of vaccination was extracted from SYSVAK and added to the MSIS dataset. After this, the PIN was deleted which anonymised the data used for analysis.

In order to measure VE, the exposure of interest was vaccination against influenza A(H1N1)pdm09. Since previous studies differ on the time needed to achieve full immunity against influenza after vaccination [8-10], we considered two different thresholds for immunity in our study: eight or more days and 15 or more days after vaccination. The outcome of interest was laboratory-confirmed influenza A(H1N1)pdm09. A case was defined as "a person with influenza A(H1N1)pdm09 infection confirmed by laboratory using reverse transcription-polymerase chain reaction (RT-PCR), registered in MSIS". Vaccine effectiveness (VE) of the pandemic influenza vaccine was measured with the following formula [9]:

$$\frac{\text{VE}=\text{1}-\text{Cumulative Incidence of influenza A}\left(\text{H1N1}\right)\text{pdm}0\text{9 in vaccinated population}}{\text{Cumulative Incidence of influenza A}\left(\text{H1N1}\right)\text{pdm}0\text{9 in unvaccinated population}}$$

As the number of cases and the number of persons vaccinated changed through the pandemic period, we measured VE overall and by week starting from week 45, when considering 15 days or more as the immunity threshold and from week 44 when considering eight days or more. People who had already had laboratory-confirmed influenza were removed from the denominator in the following weeks.

We extracted the aggregated total number of people vaccinated by week from SYSVAK. We obtained the total number of the Norwegian population as of October 2009 from Statistics Norway http://www.ssb.no.

We describe in more detail those individuals who had laboratory-confirmed influenza in spite of vaccination at least eight or 15 days earlier (vaccine failures).

## Results

When considering 15 days or more after vaccination as the threshold of immunity, VE by week ranged from 77% (95%CI 64%-86%) in week 48 to 96% (95%CI 71%-99%) in week 50. In week 51, VE was 100% since there were no vaccinated laboratory-confirmed cases reported (Table 1). When we estimated VE considering eight days or more after vaccination as the threshold, VE by week ranged from 73% (95%CI 63%-80%) in week 45 to 94% (95%CI 58%-99%) in week 51 (Table 2).

**Table 1:** Estimated effectiveness of the pandemic influenza vaccine during the main wave of the influenza pandemic in Norway.

Week	45	46	47	48	49	50	51	52	53
*Vaccinated population*									

No of cases	1	14	19	17	2	1	0	1	3

Population	32,766	266,278	501,115	698,006	873,629	1,105,763	1,397,506	1,675,354	1,891,980

Incidence(per 100,000)	3.05	5.26	3.79	2.44	0.23	0.09	0.00	0.06	0.16

*Unvaccinated population*									

No of cases	2579	1612	866	453	187	84	34	16	12

Population	4,811,608	4,575,516	4,339,053	4,141,277	3,965,184	3,732,861	3,441,033	3,163,151	2,946,508

Incidence(per 100,000)	53.60	35.23	19.96	10.94	3.72	2.25	0.99	0.51	0.41
*Vaccine effectiveness*									

Point estimate	94%	85%	81%	77%	95%	96%	100%	88%	61%

95% confidence interval	60%-99%	75%-90%	70%-88%	64%-86%	64%-99%	71%-99%	-	11%-92%	-37%-89%

Estimated effectiveness of AS03-adjuvanted monovalent vaccine during the main wave (weeks 45-53, 2009) of the influenza pandemic in Norway Vaccinees were considered immune 15 days and later after vaccination.

**Table 2:** Estimated effectiveness of the pandemic influenza vaccine during the main wave of the influenza pandemic in Norway.

Week	44	45	46	47	48	49	50	51	52	53
*Vaccinated population*										

No of cases	3	40	45	31	21	7	5	1	1	3

Population	32,766	266,276	501,087	697,952	873,561	1,105,676	1,397,413	1,675,256	1,891,882	1,891,654

Incidence(per 100,000)	9.16	15.02	8.98	4.44	2.40	0.63	0.36	0.06	0.05	0.16

*Unvaccinated population*										

No of cases	2683	2540	1,581	854	449	182	80	33	16	12

Population	4,811,608	4,575,412	4,338,021	4,139,530	3,963,036	3,730,451	3,438,525	3,160,597	2,943,937	2,919,148

Incidence(per 100,000)	55.76	55.51	36.45	20.63	11.33	4.88	2.33	1.04	0.54	0.41

*Vaccine effectiveness*										

Point estimate	84%	73%	75%	78%	79%	87%	85%	94%	90%	62%

95% confidence interval	50%-95%	63%-80%	67%-82%	69%-85%	68%-86%	72%-99%	62%-94%	58%-99%	27%-99%	-34%-90%

Estimated effectiveness of AS03-adjuvanted monovalent vaccine during the main wave (weeks 44-53, 2009) of the influenza pandemic in Norway. Vaccinees were considered immune eight days and later after vaccination.

**Figure 1:**
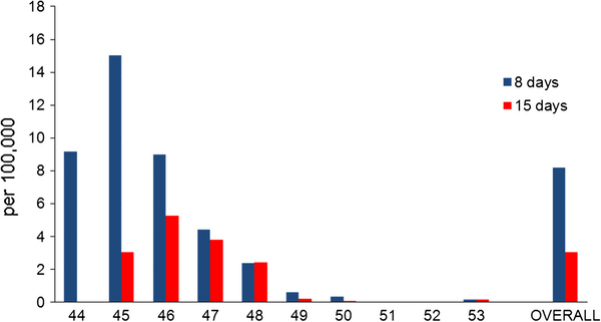
**Rates of vaccine failures per week and overall during the main wave of the influenza pandemic in Norway**. Rates of vaccine failures (per 100,000 vaccinated) per week and overall of the AS03-adjuvanted monovalent vaccine during the main wave (weeks 45-53, 2009) of the influenza pandemic in Norway. Blue bars indicate immunity as eight days and later after vaccination; red bars as 15 days and later.

A total of 157 individuals were considered vaccine failures, i.e. they had onset of symptoms of pandemic influenza eight days or more after vaccination (8.4 per 100,000 vaccinated). When defining vaccine failure as those who had onset 15 days or more after vaccination, the number of vaccine failures decreased to 58 (3.0 per 100,000 vaccinated). There were slightly more females than males: 33 (52%) when using 15 days or more as the immunity threshold and 81 (57%) when using eight days. Most of the vaccine failures occurred during the first weeks of the vaccination campaign. After week 46, the proportion of failures decreased gradually to become minimal during the last weeks of the year (Figure 1).

**Figure 2:**
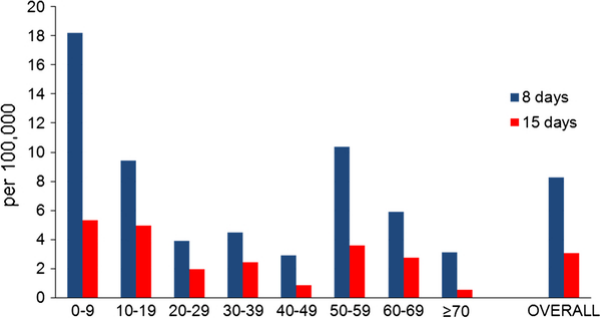
**Rates of vaccine failures by age group and overall during the main wave of the influenza pandemic in Norway**. Rates of vaccine failures (per 100,000 vaccinees) by age group and overall of the AS03-adjuvanted monovalent vaccine during the main wave (weeks 45-53, 2009) of the influenza pandemic in Norway. Blue bars indicate considering immunity eight days and later after vaccination; red bars 15 days and later.

The highest proportion of vaccine failures was observed among persons less than 10 years of age: 19 cases (33% of all vaccine failures) when considering the threshold 15 days and 65 cases (41%) when considering eight days. When moving the threshold from 15 to eight days the largest increase of vaccine failures was observed in the 0-9 years age group (increasing from 5.3 to 18.2 per 100,000 vaccinated), among those aged 50-59 years (increasing from 3.6 to 10.4 per 100,000 vaccinated) and those older than 70 years (increasing from 0.5 to 3.1 per 100,000 vaccinated) (Figure 2).

## Discussion

Pre-existing national health registries created for surveillance purposes allowed us to estimate VE and describe vaccine failures during the 2009-2010 influenza pandemic. Our results show that vaccination with an AS03-adjuvanted monovalent vaccine against A(H1N1)2009 conferred a very high protective effectiveness against laboratory confirmed pandemic influenza in Norway, regardless of immunity threshold chosen.

We measured VE by week in order to take account of the changes in the denominators over time as when more people were vaccinated. An overall calculation with fixed denominators would have yielded VE of 99% and 97% with 15 and eight days immunity thresholds, respectively. This underlines that, in specific scenarios, such as a pandemic situation where a vaccine is not distributed simultaneously to the whole population, VE should always be estimated taking into account time in order to avoid an overestimated measurement of the protection provided by the vaccine. Controlling by time has already shown to be useful when estimating VE in the pandemic in other published studies [10]. Together with time, age is a critical factor when determining VE. However, due to the low number of influenza cases notified per week in each age-group, we were not able to adjust our VE estimates by age.

There are some intrinsic characteristics of the national health registries used in our study that should be taken into account when interpreting our results. MSIS does not include all pandemic influenza cases that occurred in Norway, since at the time when the vaccination campaign began, mainly persons with severe ILI symptoms or those that belonged to a risk group were sampled. This means that the laboratory-confirmed cases notified in MSIS are not a random sample of all the cases occurring in the Norwegian population. If the likelihood of having laboratory-confirmed influenza and thus be notified to MSIS, was associated with vaccination status, our VE estimate could be biased. It should also be noted that not all vaccinations were notified to SYSVAK. Overall, the number of vaccinated population shown in Table 1 and Table 2 is expected to be around 10% higher, leading to an even lower incidence among those vaccinated. With other factors being equal, this effect alone will have led to an underestimation of the VE.

This study is also subject to other potential biases. Firstly, at the beginning of the pandemic period only groups at risk were recommended to be vaccinated. Patients with underlying conditions, like immunity disorders, may have had a lower response to the vaccine. This would result in an underestimation of the VE. This bias is known as confounding by indication. Our results may reflect this, since the proportion of vaccine failures during the first weeks after the vaccination campaign started is the highest during the whole period.

Secondly, the opposite effect, an overestimation of VE can be seen if healthy people were more likely to be vaccinated. This situation is known as healthy vaccinee confounding and may have occurred in the period when vaccination was recommended to the general population and most of the people in the risk groups had already been vaccinated. There are some indications of this bias in our results since the VE estimate improved as the vaccination campaign moved on.

Thirdly, clinicians may have been less likely to test for influenza in patients known to be vaccinated, thinking that influenza was an unlikely cause of their symptoms. Due to this potential diagnostic bias, vaccine failures may have been missed and VE overestimated. However, the NIPH encouraged clinicians to document suspected vaccine failures by testing in order to minimize this bias.

Finally, as the campaign developed, more and more persons were immune due to vaccination or natural immunity following infection. This probably led to some degree of herd protection, which may also have had an effect on the measured VE.

Our results are in concordance with findings of published meta-analyses of randomized trials of influenza vaccines [11,12]. They are also consistent with the conclusions of previous pandemic influenza VE studies conducted in other European countries [9,13-16] and Canada [17], where it was concluded that, overall, the pandemic influenza vaccine was highly effective in preventing laboratory-confirmed infection with pandemic influenza A(H1N1)pdm09. Our study also supports findings in of previous preliminary studies conducted in Norway during the pandemic period [18].

Regardless the high VE measured, our results highlight specific age groups that seemed to be less protected by the vaccine and in need of more time to develop immunity. Special attention should be paid to those less than 10 years of age due to high number of people involved. However, in order to be able to recommend specific precautions after vaccination in this age group, further studies controlling for potential confounding factors (like underlying diseases) should be carried out. The nature of our study, using already available register based information, did not allow us to control for such factors.

We used two existing registries for this study. MSIS, the infectious disease register, was augmented during the pandemic by inclusion of laboratory-confirmed influenza A(H1N1)pdm09 as notifiable disease on a case based level. This mainly served surveillance purposes and for informing the public health response, but the data could also be used for evaluation of the interventions put in place, as shown in this study. The cost of the augmentation was small as already existing procedures and infrastructure was used. SYSVAK, the immunisation register, was augmented by including pandemic vaccination. The purpose of this was both to monitor the execution of the vaccination campaign and to prepare for follow-up studies. A web-based platform was introduced and linked to the national population register to facilitate easy entry of each vaccination by the vaccinators. The time needed for entry of one vaccination was less than 30 seconds. Because of the high number of vaccinations, however, the total time spent on registrations was considerable.

## Conclusions

We have shown that available health registries with surveillance data on infectious diseases and vaccination made it feasible to estimate VE in a simple and rapid way. We encourage local, regional and national public health authorities in other countries to set up such registries. It is also important to consider including information on underlying diseases in registries already existing, in order to make it feasible to conduct more complete VE estimations by controlling for potential confounding factors that may affect the results obtained.

## Competing interests

The authors declare that they have no competing interests.

## Authors' contributions

PAa, BF, MRB and KB conceived of the study. PAa, KB and BGH participated in its design. BGH analysed the information and drafted the manuscript. All authors participated in manuscript writing and revision. All authors read and approved the final manuscript.

## Pre-publication history

The pre-publication history for this paper can be accessed here:

http://www.biomedcentral.com/1471-2334/12/63/prepub

## References

[B1] Folkehelseinstitutet Rapport om scenarier for pandemien og andre influensaepidemier i 2010-2011 (Norwegian Institute of Public Health: Report on scenarios for pandemic influenza and other influenza epidemics in 2010-2011) Norwegian http://www.fhi.no/dokumenter/3c807895b6.pdf

[B2] European Medicines Agency Pandemrix. Summary for the public http://www.ema.europa.eu/ema/index.jsp?curl=pages/medicines/human/medicines/000832/human_med_000965.jsp&murl=menus/medicines/medicines.jsp&jsenabled=true

[B3] European Centre for Disease Prevention and Control The 2009 A(H1N1) pandemic in Europe. A review of the experience. Special report. Stockholm 2010

[B4] Folkehelseinstituttet Faktahefte om vaksine mot pandemisk influenza. (Norwegian Institute of Public Health: Facts about vaccine against pandemic influenza.), Norwegian http://www.fhi.no/dav/f81e5aec12.pdf

[B5] Folkehelseinsitituttet Rapport om anbefalt rekkefølge for vaksinering mot ny influensa A(H1N1), 16. september 2009. (Norwegian Institute of Public Health: Report on the recommendations for vaccination against the new influenza A (H1N1), 16 September 2009), Norwegian http://www.fhi.no/dokumenter/ec53d5ef4f.pdf

[B6] Folkehelseinsitituttet Rapport nummer to om vaksinasjon rekkefølge, versjon 2 23.10.2009. (Norwegian Institute of Public Health: Report number two on the vaccination recommendations, version 2 23.10.2009), Norwegian http://www.fhi.no/dokumenter/f332d0ae39.pdf

[B7] Direktoratet for samfunnssikkerhet og beredskap Rapport. Ny influenza A(H1N1) 2009. Gjennomgang av erfaringene I Norge.; 2010. (Norwegian Directorate for Civil Protection and Emergency Planning: Report. New influenza A(H1N1) 2009. Review of the experiences in Norway; 2010.), Norwegian

[B8] European Centre for Disease Prevention and Control Protocol for cohort database studies to measure influenza vaccine effectiveness in the European Union and European Economic Area Member States. Technical document 2009

[B9] Valenciano M Kissling E Cohen J-M Oroszi B Barret A-S Rizzo C Nunes B Pitigoi D Larrauri Cámara A Mosnier A Horvath JK O'Donnell J Bella A Guiomar R Lupulescu E Savulescu C Ciancio B Kramarz P Moren A Estimates of Pandemic Influenza Vaccine Effectiveness in Europe, 2009-2010: Results of Influenza Monitoring Vaccine Effectiveness in Europe (I-MOVE) Multicentre Case-control Study PLoS Med 2011 8 1 e1000388 10.1371/journal.pmed.1000388 21379316 PMC3019108

[B10] Emborg HD Grove Krause T Hviid A Simonsen J Mølbak K Effectiveness of vaccine against pandemic influenza A/H1N1 among people with underlying chronic diseases: cohort study, Denmark, 2009-10 BMJ 2012 344 d7901 22277542 10.1136/bmj.d7901

[B11] Manzoli L De Vito C Salanti G D'Addario M Villari P Ioannidis J Meta-Analysis of the Immunogenicity and Tolerability of Pandemic Influenza A 2009 (H1N1) Vaccines PLoS ONE 2011 6 9 e24384 10.1371/journal.pone.0024384 21915319 PMC3167852

[B12] Osterholm M Kelley N Sommer A Belongia E Efficacy and effectiveness of influenza vaccines: a systematic review and meta-analysis Lancet Infect Dis 2012 12 36 44 10.1016/S1473-3099(11)70295-X 22032844

[B13] Hardelid P Flemng D McMenamin J Andrews N Effectiveness of pandemic and seasonal influenza vaccines in preventing pandemic influenza A(H1N1) 2009 infection in England and Scotland 2009-2010 Euro Surveill 2011 16 2 pii=19763 21251487

[B14] Simpson CR Ritchie LD Robertson C Sheikh A Effectiveness in pandemic influenza - primary care reporting (VIPER): an observational study to assess the effectiveness of the pandemic influenza A (H1N1)v vaccine (Scotland) Health Technol Assess 2010 14 313 346 20630126 10.3310/hta14340-05

[B15] Wichmann O Stöcker P Poggensee G Altmann D Pandemic Influenza A (H1N1) 2009 breakthrough infections and estimates of vaccine effectiveness in Germany 2009-2010 Euro Surveill 2010 15 18 pii 1956 20460094

[B16] Ortqvist Å Berggren I de Jong B Svenungsson B Effectiveness of an adjuvanted monovalent vaccine against the 2009 pandemic strain of Influenza A(H1N1)v, in Stockholm county, Sweden Clin Infec Dis 2011 52 1203 1211 10.1093/cid/cir182 21507917

[B17] Van Buynder PG Dhaliwai JK Van Buynder JL Minville-LeBlanc M Protective effect of single dose adjuvanted pandemic influenza vaccine in children Influenza Other Respir Viruses 2010 4 171 178 10.1111/j.1750-2659.2010.00146.x PMC5964543 20629771

[B18] Mamelund SE Storsæter J Haugen IL Ege MS Bergsaker MA Clinical efficiency after one dose of Pandemrix in Norway BMJ Rapid Response 2009 http://www.bmj.com/rapid-response/2011/11/02/clinical-efficiency-after-one-dose-pandemrix-norway

